# POSITIVE EMOTION LANGUAGE PREDICTS IMPROVING DEPRESSION TRAJECTORIES AFTER DEMENTIA CAREGIVING ENDS

**DOI:** 10.1093/geroni/igad104.3732

**Published:** 2023-12-21

**Authors:** Jenna Wells, Julian Scheffer, Suzanne Shdo, Claire Yee, Katherine Possin, Robert Levenson

**Affiliations:** Cornell University, Ithaca, New York, United States; University of California, Berkeley, Berkeley, California, United States; University of California, Berkeley, Berkeley, California, United States; Mayo Clinic, Scottsdale, Arizona, United States; University of California, San Francisco, San Francisco, California, United States; University of California, Berkeley, Berkeley, California, United States

## Abstract

There are striking differences among caregivers of people with dementia (PWDs) in how their health and well-being changes during active caregiving; however, less is known about these differences after caregiving has ended. One important factor influencing caregiver health is the emotional quality of the caregiver-PWD relationship. The present study evaluated caregivers’ emotional connection to the PWD (during caregiving) and associations with caregivers’ depression both concurrently (N = 326 active caregivers) and longitudinally (n = 91 former caregivers). Active caregivers described a recent time they felt connected to the PWD, and their responses were transcribed verbatim. Caregivers’ emotional connection to the PWD was operationalized by summing the number of positive and negative emotion words from the transcripts using text analysis software and dividing each figure by the total number of words in the response. Results indicated that neither positive nor negative emotional language was significantly correlated with caregivers’ concurrent depression. However, two-wave latent change score modeling revealed that caregivers’ greater positive emotional language (adjusting for caregivers’ baseline depression and negative emotional language) predicted steeper declines in depression (i.e., improvements in depression trajectories) after the death of the PWD (ß = -.11, SE(ß) = 0.04, p = .033). Negative emotional language was not significantly associated with changes in caregivers’ depression. This finding highlights positive emotion expressed by caregivers when describing their connection with the PWD as a potential resilience factor and underscores the important role that positive emotional qualities of the caregiving relationship play in improving caregivers’ mental health after caregiving ends.

